# Performance of photocatalytic oxidation of tetracycline in aqueous solution by TiO_2_ nanofibers

**DOI:** 10.1186/2052-336X-11-24

**Published:** 2013-08-02

**Authors:** Allahbakhsh Javid, Simin Nasseri, Alireza Mesdaghinia, Amir hossein Mahvi, Mahmood Alimohammadi, Rouhollah Mehdinavaz Aghdam, Noshin Rastkari

**Affiliations:** 1 Department of Environmental Health Engineering, School of Public Health, Tehran University of Medical Sciences, Tehran, Iran; 2 Center for Water Quality Research, Institute for Environmental Research, Tehran University of Medical Sciences, Tehran, Iran; 3 Center for Solid Waste Research, Institute for Environmental Research, Tehran University of Medical Sciences, Tehran, Iran; 4 Department of Nanotechnology, Engineering Research Institute, Tehran, Iran; 5 Center for Air Pollution Research, Institute for Environmental Research, Tehran University of Medical Sciences, Tehran, Iran

**Keywords:** Antibiotic, Photocatalytic, Response surface methodology, TiO_2_ nanofibers, Tetracycline

## Abstract

The presence of pharmaceutical compounds in water and soil has become an environmental concern. The aim of this study was to evaluate the performance of TiO_2_ nanofiber in the oxidation of the antibiotic tetracycline. TiO_2_ nanofiber was fabricated by electro-spinning method, and then was calcined at 560°C for 2 h. Central composite design (CCD) statistic model was used to optimize tetracycline concentration, time and pH for TiO_2_ catalyst. A tubular Pyrex glass reactor with diameter of 15 cm and height of 30 cm was designed and a 125W Philips HPLN lamp (UV, *λ* > 254 nm) was used as light source. Samples were measured by high-performance liquid chromatography (HPLC). Equation of model suggests a direct relationship between pH and time with efficiency of tetracycline removal. The observations indicated that time is the most significant (scaled estimate = + 28.04) parameter in efficiency of tetracycline removal. The application of response surface methodology yielded the equation of Y = 65.82 + 5.74 pH + 28.04 time + (−3.07)(pH)^2^ + (−6.6)(time)^2^, with R^2^ = 0.986 which represents good reliability of model. Based on the response surface plots optimum conditions for degradation of tetracycline with maximum efficiency of around 95% was attained. These conditions are as follow; concentration: 50 mg/l, pH= 8.3, time= 15 min.

## Introduction

In recent years the use of antibiotics for veterinary and medical purposes has been widely increased (100,000–200,000 ton/year) and therefore the possibility of water contamination with these compounds has increased [1]. The presence of antibiotics in water and soil can cause some allergies and toxicity [2,3]. Additionally, a few studies on the presence of antibiotic residues in surface water, ground water, sea and drinking water have been reported [4-6]. In addition, the presence of antibiotic residues in wastewater treatment plant effluents and hospital wastewaters have been detected [7-9]. Tetracycline (TC) is used mostly in aquaculture and veterinary medicine. The amount of drug use in animals is higher, for example, in poultry: 20–300 mg per kg of body weight is consumed. Like other antibiotics, tetracycline is discharged into water resources through drug manufacturing wastewater, disposal of non-consumable compounds, expired drugs containing tetracycline and from animal and agricultural wastes [10,11].

Various technologies are used to degrade tetracycline; one group of these technologies is comprised of Advanced Oxidation/Reduction Processes (AO/RPs) which have potential for degradation of contaminated waters by antibiotics. When these technologies operate, they produce radicals that can react and destroy contaminants in the water. AO/RP methods typically generate hydroxyl radicals (OH^•^) which include O_3_/UV, O_3_/H_2_O_2_, TiO_2_/UV, ZnO/UV, H_2_O_2_/UV, and UV/Photo-Fenton [12].

*As in recent studies reported*, heterogeneous photocatalysis for TC degradation by TiO_2_ has been cost-effective and environmentally sustainable treatment alternative. TiO_2_ is one of most suitable photocatalysts for water treatment, because of its high photocatalytic activity, good chemical and biological stability, high energy efficiency, minimum waste production, relatively low cost, and non-toxicity [13]. Several studies have investigated photocatalytic oxidation of the antibiotic tetracycline on TiO_2_ particles and TiO_2_ coated onto the fixed bed [13-16]. In a report the effect of UV type (UV at 254 and 365 nm and solarium device at 300–400 nm) with TiO_2_ nanoparticle on TC degradation was studied [16]. Also, recently a response surface model for predicting the efficiency of TC degradation by TiO2 and ZnO was developed using The Central Composite Circumscribed (CCC) statistic model to optimize the amount of catalyst and the pH of the reaction [15]*.*

In that work, two photocatalitic factors of catalyst and pH were used in the CCC design. In this study, for the first time the effects of three factors were studied by using Central Composite Design (CCD) and TiO_2_ nanofiber was used as catalyst TC degradation.

The performance of TiO_2_ nanofiber in the oxidation of antibiotic tetracycline has been evaluated. The Response Surface Methodology (RSM) was used to optimize pH, tetracycline concentration and time. The degradation processes were monitored by measuring tetracycline disappearance.

## Material and methods

### Preparation of precursor solution for electrospinning

Polyvinylpyrrolidone (PVP) (MW=1,300,000 g/mol purchased from Aldrich Co., code: 437190) was dissolved in the mixture of ethanol/acetic acid (2.7:1w:w; Merck Co.) at 60°C for 3 h. Titanium isopropoxide (TTIP) (Aldrich) was added to the PVP solution with vigorous stirring for 6 h. A homogeneous solution including 6.5 wt. % PVP and 10 wt. % TTIP was prepared. The obtained mixed solution was used as the working fluid for electrospinning [17].

### Fabrication of TiO_2_ nanofibers

**Figure 1:**
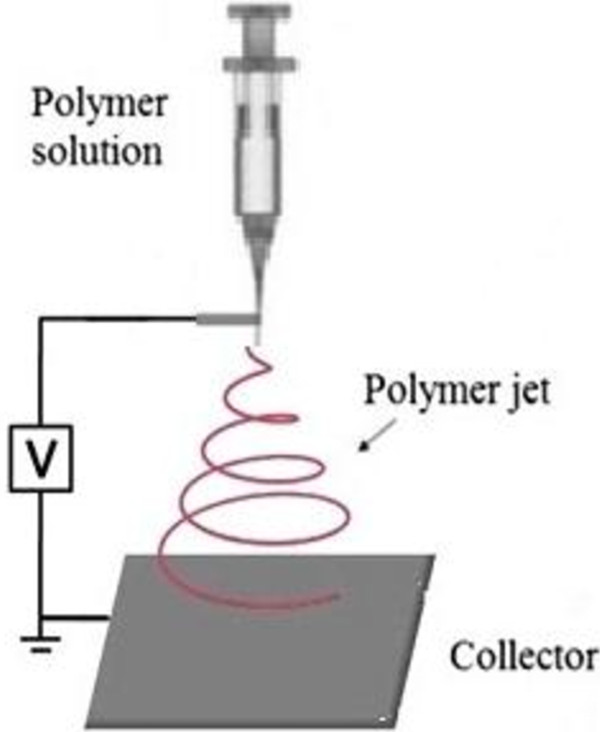
Schematic diagram of electro spinning technique.

The prepared precursor solution (volume: 10 mL) was applied to a droplet of PVP/TTIP precursor solution at the tip of a syringe needle with 0.4 mm inner diameter. The flow rate of the solution was 9 μL/min controlled by a syringe pump. A voltage of 18 kV was applied between the needle and the aluminum foil, which was placed at 20–25 cm from the syringe tip and used to collect the nanofibers. A schematic diagram of the electrospinning apparatus is shown in Figure 1. After electrospinning, the prepared nanofibers were calcined at 560°C for 2 h. Scanning Electron Microscope (SEM), from Philips Co. (Holland), was used for measuring the fiber diameters and observation of fiber morphologies. TiO_2_ nanofibers were characterized by X-ray diffraction (X-ray diffractometer, Philips, PW 1800).

### Response surface methodology

The Central Composite Design (CCD) statistic model was used in order to optimize the amount of TC concentration, time and pH of the reaction for TiO_2_ catalyst. The optimization procedure for photocatalytic reactions consisted of an experimental design to identify the importance of each variable involved in the reaction with a minimum set of experiments. The variables under study were codified for their higher and lower values (+1, -1), simultaneously changed in a defined range and processed with the adequate statistical software. Multivariate analysis was used to determine the weight and interaction between the variables, obtaining a polynomial and a graphical representation, or response surface, which provides the region where the optimized reaction occurs [17]*.* In the present study, The variables included concentration of TC, reaction time and pH at five coded levels, -α, -1, 0, +1, +α (the number of is 1.68), as shown in Table 1 in which the actual levels of variables for CCD experiments are also given. The CCD included 20 experimental trials with five trials as replication of the central points.

**Table 1:** The variables and their levels for the central composite experimental design

**Independent variable**	**Coded levels**				
	**-α**	**−1**	**0**	**+1**	**+α**
**Concentration (mgL**^**-1**^**)**	7.95	25	50	75	92
**pH**	2	4	7	10	12
**Time (min)**	0	3	7.5	12	15

### Photocatalytic activity test

A tubular Pyrex glass reactor with diameter of 15 cm and height of 30 cm was used in this study. The reaction was always maintained under cool condition by circulating water (all photocatalytic experiments were performed at ambient temperature). A 125W Philips HPLN lamp (UV, *λ* ≥ 254 nm) was used for light sources. To avoid the effects of the sunlight, the reactor was covered with aluminum foil. UV light lamp was encapsulated in a quartz jacket and positioned in the middle of the reactor. In a typical experiment, catalyst (TiO_2_ nanofiber) was added into TC solution with an initial concentration of (500 mg/L).

### Analytical methods

The samples were taken by syringe and filtered through 0.45 μm membranes at pre-selected time intervals, and then measured using high-performance liquid chromatography (HPLC). HPLC consisted of a Knauer LPG pump, an EZ-chrom HPLC system manager program and a UV detector (k-2500). The UV–detector was set at the maximum absorption wavelength for 365 nm. Aliquots of 100 μL were injected manually using a model SGE injection valve (SGE. Australia). A column of MZ-analysentechnik ODS-3 C18 (4.6 mm×250 mm) packed with 5 μm spherical particles was used for separation. An acetonitrile/0.01 mol/L aqueous oxalic acid (31:69, v/v) mixture was used as mobile phase at 30°C with a constant flow rate of 1.0 mLmin^−1^[18]. The TC retention time in HPLC analysis was 3.83 min.

## Results and discussion

### Characterizations of synthesized TiO_2_ nanofiber

**Figure 2:**
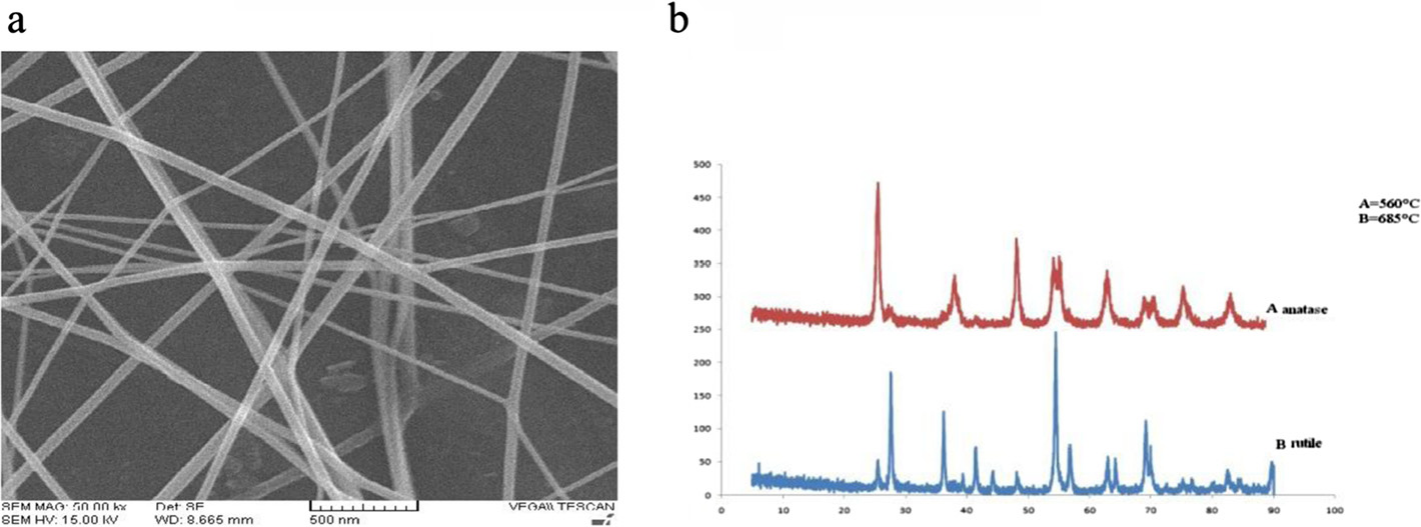
**Characteristic of fabricated TiO**_**2 **_**(SEM, TG-DTA, XRD analysis): Typical SEM image of nanofibers after calcinations (a).** X-ray diffraction patterns of electro spinning synthesized TiO_2_nanofiberscalcined at 560°C and 685°C for 2 h in an air atmosphere **(b)**.

Figure 2a shows a typical SEM image of the synthesized nanofibers. The image was taken from a sample containing 6.5% PVP and 10% TTIP, distance= 20 cm, applied voltage=18 kV and flow rate=0.6 ml/h. This image shows that diameter of nanofiber is between 29–90 nm. Figure 2b shows X-ray diffraction patterns of the synthesized TiO_2_ nanofiber after calcinations at 560°C and 685°C for 2 h under air atmosphere. As shown in Figure 2b the formation of pure anatase TiO_2_ phase attained after calcinations at 560°C. Increasing the temperature to 685°C led to formation of rutile TiO_2_ as well. Figure 2a displays SEM images of TiO_2_ nanofibers calcined at 560°C in an air atmosphere for 2 h.

### Optimization of the reaction

Central Composite Design matrix of three variables in coded and natural units with the observed responses is shown in Table 2. A typical response surface function for three input variables is in the form of the following equation:

**Table 2:** Experimental design and results of the central composite design

**Run**	**X**_**1**_	**X**_**2**_	**X**_**3**_	**Con. (mg/L)**	**pH**	**Time (min)**	**Efficiency (%)**
**1**	0	0	0	50	7	7.5	65.8
**2**	1	1	−1	75	10	3	40.0
**3**	0	0	0	50	7	7.5	65.4
**4**	1	−1	1	75	4	12	76.6
**5**	−1	−1	1	25	4	12	75.0
**6**	0	0	0	50	7	7.5	66.2
**7**	−1.68	0	0	7.95	7	7.5	59.0
**8**	0	0	+1.68	50	7	15	95.0
**9**	1	−1	−1	75	4	3	15.2
**10**	1	1	1	75	10	12	82.6
**11**	0	0	0	50	7	7.5	65.8
**12**	−1	1	1	25	10	12	90.0
**13**	−1	−1	−1	25	4	3	15.0
**14**	0	+1.68	0	50	12	7.5	62.5
**15**	0	0	−1.68	5	7	0	0.0
**16**	+1.68	0	0	92	7	7.5	58.1
**17**	0	0	0	50	7	7.5	66.0
**18**	0	0	0	50	7	7.5	65.6
**19**	−1	1	−1	25	10	3	30.8
**20**	0	−1.68	0	50	2	7.5	52.5

(1) $$\begin{array}{l} Y=b_{0}+b_{1}x_{1}+b_{2}x_{2}+b_{3}x_{3}+b_{12}x_{1}x_{2}\\ \;+b_{13}x_{1}x_{3}+b_{23}x_{2}x_{3}+b_{11}x_{1}^{2}+b_{22}x_{2}^{2}\\ \;+b_{33}x_{3}^{2} \end{array}$$

The parameters included TC concentration, pH and time. The levels of input variables are shown in Table 1.

The efficiency of correlated model was checked by the coefficient of determination (R^2^). In this case, the value of the determination coefficient was indicated as (R^2^ = 0.986). The value of the adjusted determination coefficient (Adj. R^2^ = 0.976) is a high significance of the model. The application of response surface methodology yielded the Eq. (2), which is an empirical relationship between the efficiency of TC removal and the test variables in coded unit:

(2) $$\begin{array}{l} Y=65.82+5.{74\;X}_{2}+28.0{4X}_{3}+\left(-3.07\right)X_{2}^{2}\\ \;+\left(-6.6\right)X_{3}^{2} \end{array}$$

Where Y is the response (efficiency of TC removal) and X_2_, X_3_ are the coded values of the test variables that are shown in Table 2. This equation suggests a direct relationship between pH and time with efficiency of TC removal (P < 0.05). These observations indicate that time is the most significant (scaled estimate=+28.04) parameter in the efficiency of TC removal. Except for the linear term X_2_ (pH), X_3_ (time), (P < 0.05) and quadratic term X_2_^2^ and X_3_^2^ (p < 0.05), none of the other linear, quadratic and interaction terms were statistically significant.

**Figure 3:**
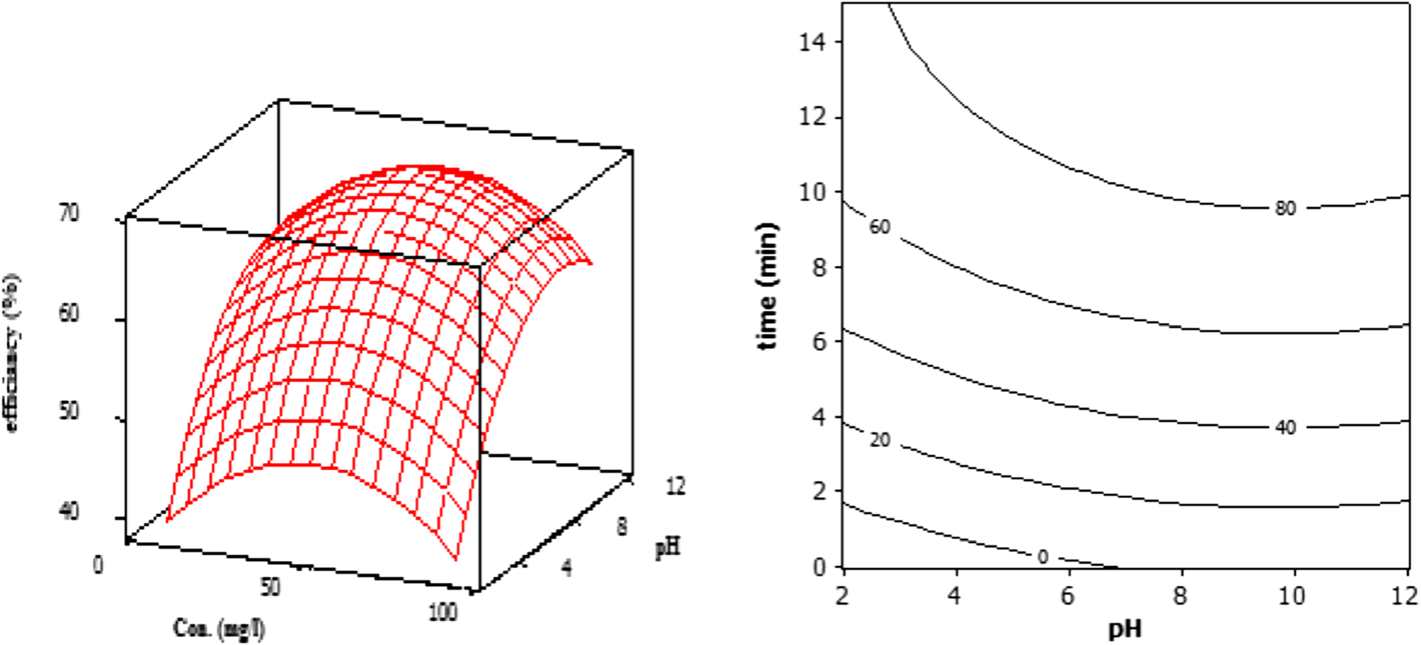
Contour plot and response surface showing the percentage of TC degradation, the effect of pH, time and concentration.

The quadratic effect of pH indicated that at both high and low pH values, the reaction will be less efficient, where this negative effect is more pronounced in acidic conditions, as shown in the response surface and contour plots (Figure 3). Under acidic pH (<3.3), both tetracycline and Titania are positively charged provoking a repulsive effect. At pH=8.7, TC shows a negative charge increasing the repulsive effect with the Titania surface, which is also partially negatively charged. At higher pH (>9.7) the repulsive effect increases because TC shows a double negative charge [15].

**Figure 4:**
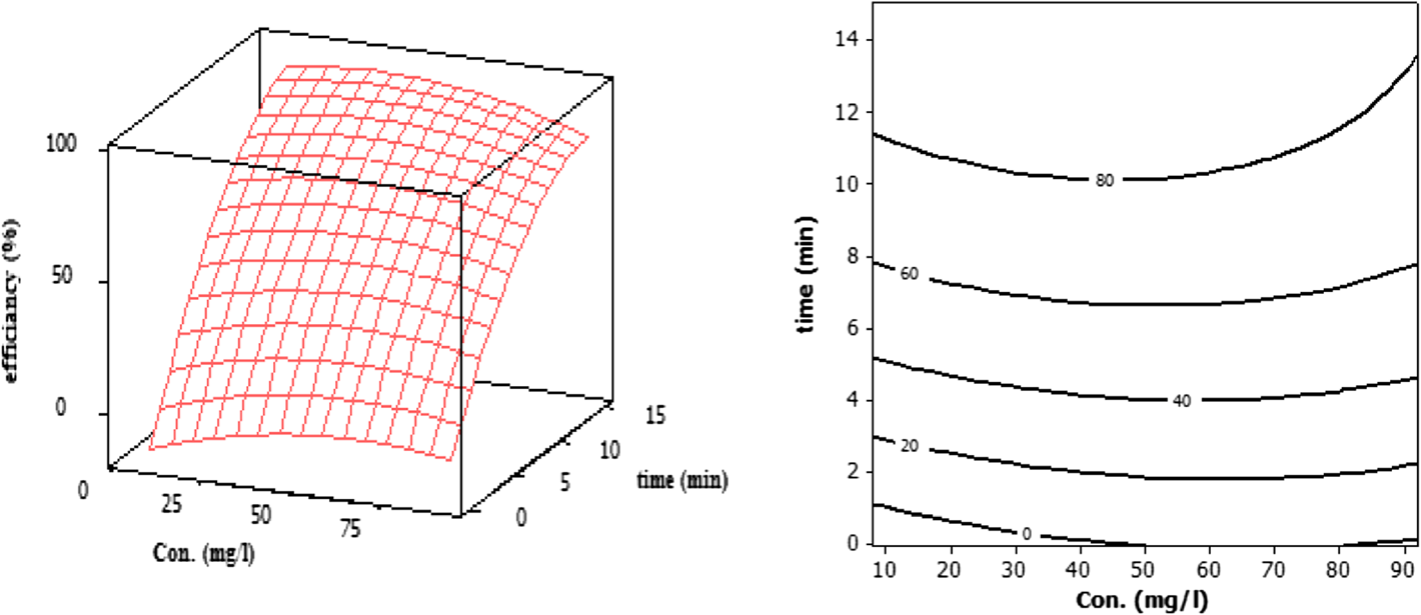
Contour plot and response surface showing the percentage of TC degradation, the effect of time, pH and concentration.

**Figure 5:**
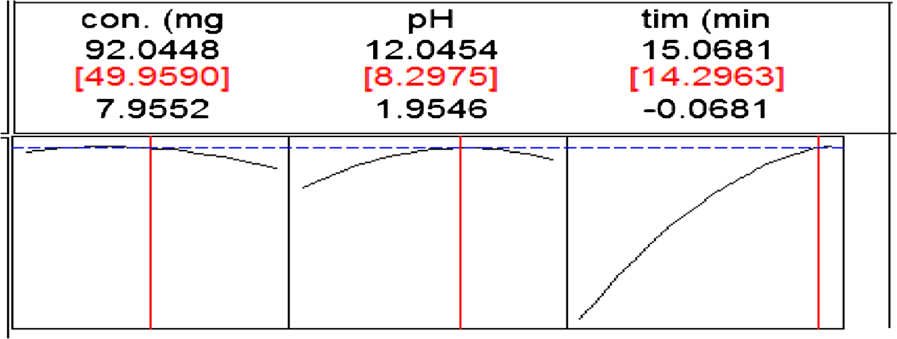
Prediction profiler and desired conditions for efficiency of TC removal about 95%.

The predicted values of efficiency of TC removal based on the range of time were estimated as contour and response surface plots (Figure 4). In this contour and response surface plot, the effect of two variables on efficiency of TC removal is shown. Based on Response surface plots optimum conditions for degradation of TC with maximum efficiency around 95% was achaived. These conditions are as follows; concentration=50 mg/l, pH=8.3, time=15 min (Figure 5). The optimal pH for degradation of TC 8.7 has been reported by Rodrigo et al. [15].

Degradation of TC in the UVC/TiO_2_ nanofiber reactor in different pH_S_ in all concentrations followed first order kinetics model (Table 3). Most studies previously reported the kinetics of photolysis of pollutants to be first order [14,16]*.*

**Table 3:** **Kinetic order degradation of TC at various pH, subjected to UVC/TiO**_**2 **_**nanofiber reactor**

**C0 (mg/l)**	**pH**	**k (1/min)**	**Reaction order**
**75**	10	0.2531	First order
**75**	7	0.1378	First order
**75**	4	0.1568	First order
**50**	10	0.2296	First order
**50**	7	0.1250	First order
**50**	4	0.1423	First order
**25**	10	0.2055	First order
**25**	7	0.1119	First order
**25**	4	0.1273	First order

## Conclusion

The application of response surface methodology yielded the equation of Y = 65.82 + 5.74 pH + 28.04 time + (−3.07)(pH)^2^ + (−6.6)(time)^2^ with R^2^ = 0.986 which represents good reliability of model. The effect of various parameters on degradation efficiency of tetracycline was studied via concentration of TC analysis by HPLC. For instance with increasing pH value from 4 to 10, the degradation efficiency of tetracycline increased from 75.8% ± 0.8 to 86.3% ± 3.7 (time = 15 min). Based on Response surface plot, optimum conditions for degradation of TC with maximum efficiency around 95% were attained. These conditions are as follow: concentration= 50 mg/l, pH= 8.3, time= 15 min.

## Competing interests

The authors declare that they no competing interests.

## Authors’ contributions

AJ was the main investigator, collected the data, performed the statistical analysis, and drafted the manuscript. SN and AM supervised the study. AHM, MA, RMA and NR were advisors of the study. All authors read and approved the final manuscript.
